# Disabled-2 Determines Commitment of a Pre-adipocyte Population in Juvenile Mice

**DOI:** 10.1038/srep35947

**Published:** 2016-10-25

**Authors:** Wensi Tao, Robert Moore, Yue Meng, Toni M. Yeasky, Elizabeth R. Smith, Xiang-Xi Xu

**Affiliations:** 1 Department of Cell Biology and Sylvester Comprehensive Cancer Center, University of Miami Miller School of Medicine, Miami, FL, 33136, USA; 2 Graduate Program in Molecular Cell and Developmental Biology, University of Miami Miller School of Medicine, Miami, FL 33136 USA

## Abstract

Disabled-2 (Dab2) is a widely expressed clathrin binding endocytic adaptor protein and known for the endocytosis of the low-density lipoprotein (LDL) family receptors. Dab2 also modulates endosomal Ras/MAPK (Erk1/2) activity by regulating the disassembly of Grb2/Sos1 complexes associated with clathrin-coated vesicles. We found that the most prominent phenotype of Dab2 knockout mice was their striking lean body composition under a high fat and high caloric diet, although the weight of the mutant mice was indistinguishable from wild-type littermates on a regular chow. The remarkable difference in resistance to high caloric diet-induced weight gain of the *dab2*-deleted mice was presented only in juvenile but not in mature mice. Investigation using Dab2-deficient embryonic fibroblasts and mesenchymal stromal cells indicated that Dab2 promoted adipogenic differentiation by modulation of MAPK (Erk1/2) activity, which otherwise suppresses adipogenesis through the phosphorylation of PPARγ. The results suggest that Dab2 is required for the excessive calorie-induced differentiation of an adipocyte progenitor cell population that is present in juvenile but depleted in mature animals. The finding provides evidence for a limited pre-adipocyte population in juvenile mammals and the requirement of Dab2 in the regulation of Ras/MAPK signal in the commitment of the precursor cells to adipose tissues.

Adipose tissue is the main site of energy storage, in a form as neutral lipids^1^,^2^. In response to excessive dietary caloric intake or starvation, the adipocytes can expand or condense in both cell size and number^3^,^4^. Excessive fat storage also leads to pathological conditions, and obesity is a pandemic afflicting modern society^5^.

Substantial understanding on the differentiation of adipocytes has been obtained from studies of cultured cells and knockout mice^6^,^7^,^8^. The transcription factor, peroxisome proliferator-activated receptor γ (PPARγ), is a critical master regulator of the adipocyte lineage^9^,^10^,^11^, and multiple signaling pathways regulate adipocyte differentiation^6^,^7^,^8^. Among these, the Ras/MAPK pathway is essential for pre-adipocyte establishment at an earlier step^12^,^13^, but its activity also antagonizes later stages of adipocyte differentiation^14^,^15^,^16^. In cell culture studies, activated MAPK (Erk1/2) reduces PPARγ expression, and also phosphorylates PPARγ, thereby preventing its nuclear accumulation and transcriptional activity^17^. Several proteins, including Pref-1, Dok1, and p62, that are involved in Ras/MAPK pathway regulation also modulate adipose development^16^,^18^,^19^.

In our current analysis of Disabled-2 (Dab2) conditional knockout mice^20^, we provide interesting clues to a role of Dab2 in the regulation of Ras/MAPK (Erk1/2) in adipocyte differentiation and adipose tissue expansion in intact animals. Dab2 is a widely expressed signaling and endocytic adaptor protein that binds clathrin and cargo proteins^21^,^22^. Dab2 also binds Grb2, an adaptor for growth factor receptors in mediating Ras/MAPK activation^23^,^24^. Dab2 regulates Grb2/Sos1 disassembly in endosomes and modulates Ras/MAPK signaling from endo-membranes but not plasma membranes^25^. Dab2 knockout is early embryonic lethal due to the failure to form an organized primitive endoderm and subsequent extraembryonic tissue defects^20^,^26^,^27^. However, *dab2* conditional knockout using Sox2-cre that restricted gene deletion to bypass extraembryonic requirement produced viable mice that are essentially depleted of Dab2 protein^20^,^28^. Dab2 null embryos have increased phospho-Erk1/2, indicating that Dab2 suppresses Ras/MAPK pathway in a physiological setting^25^. Dab2 null mice have little observable developmental abnormality or phenotypes^20^,^28^. Nevertheless, we observed that the Dab2 null mice are resistant to high fat diet-induced obesity, and uncovered its role in controlling the differentiation of a pre-adipocyte population.

## Results

### Juvenile Dab2 null mice are resistance to high calorie-induced weight gain

We produced Dab2 conditional knockout mice with an essentially complete absence of Dab2^20^ using a Sox2-cre line^29^, which allows us to bypass embryonic requirement of Dab2 and to investigate its physiological roles in intact animals. The Dab2 null mice appear mostly normal, though we observed a slight increase in serum cholesterol^20^,^30^, which is consistent with the role of Dab2 as an endocytic adaptor for the LDL receptor^31^. To investigate further the importance of Dab2 in LDL metabolism, we challenged the Dab2 null (*dab2* (fl/df);Sox2-Cre) mice with a high fat diet. However, only small perturbation in serum cholesterol level was observed, suggesting a redundant role of additional LDL receptor adaptor such as Arh^30^,^32^.

**Figure 1:**
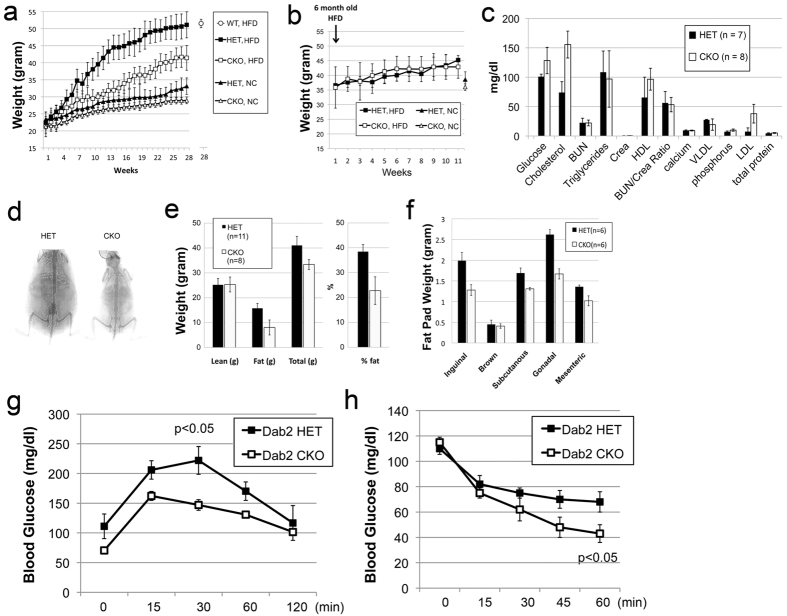
Resistance to high fat diet-induced weight gain in Dab2 conditional knockout mice. (**a**) Wild-type (WT), Dab2 Sox2-Cre conditional knockout (CKO), and heterozygous (HET) controls male mice at 7 weeks of age were placed on either normal chow (NC) or high fat diet (HFD) for additional 28 weeks. The averages of weight from 10 to 11 animals are shown with standard deviations. The weight for the WT group (n = 7) on HFD is shown for only the last time point. (**b**) Impacts of HFD on weight gain in mature mice were examined. The mice were initially fed a NC and then switched to a HFD at 6 months of age for another 11 weeks, in comparison to mice that were continued on NC (only the last time point is shown). No statistical difference was found between the two genotypes. (**c**) Blood chemistry analysis was performed on fasting *dab2* CKO and HET mice that had been fed with a HFD. The items are shown as mg/dL, except total protein that is shown as g/dL. BUN, Blood Urea Nitrogen; Crea, creatinine; LDL, low density lipoprotein; VLDL, very low density lipoprotein; HDL, high density lipoprotein. (**d**) Representative PIXI images are shown of 6-month-old *dab2* CKO and HET littermates fed a HFD. (**e**) The lean, fat, and total body masses were determined by the DEXA system and the means and standard deviations from a group of 11 HET and 8 CKO mice are presented. The difference in the percentage of body fat is statistically significant (p < 0.005) between *dab2* CKO and HET. (**f**) The fat tissue masses (inguinal, brown, subcutaneous, gonadal, and mesenteric) were determined in 6 each of the *dab2* HET and CKO male mice (p < 0.01, except brown fat). (**g**) Glucose tolerance test: Mice (6 each) were fasted for four hours and injected intraperitoneally (IP) with glucose (20% in saline) at a dosage of 2 g of glucose/kg body mass. A drop of blood (about 5 μl) was collected from tail bleeding at each time point for analysis by glucose meter. (**h**) Insulin sensitivity test: Mice (6 per group) were fasted for four hours, and then injected (IP) with insulin at a dosage of 0.6 u/kg body weight.

Unexpectedly, we observed a profound resistance to high fat diet-induced weight gain in Dab2-deficient mice, although no notable differences in weights between wild-type and *dab2* null mice were observed when fed a normal chow (Fig. 1). Following repeated observations of the effect of a high fat diet in many occasions, we specifically designed experiments to document the weight gain of Dab2 null and control mice on either normal (fat composition is 10% of total calorie) or high fat (60% fat) chow over a 6-month period, recording the weight of each animal weekly (Fig. 1a). Both male and female Dab2 null mice were resistant to high fat diet-induced weight gain. Since there were substantial weight differences between the sexes, we used only male mice in subsequent large-scale formal analyses. Also, *dab2* heterozygous littermates were used as controls for comparison with the Dab2 null mice, since we observed that *dab2* heterozygous mice were identical to wild-types in growth and high fat diet-induced weight gain.

On normal chow, the body weights of Dab2 null and control mice barely differed over a 28-week period (Fig. 1a). The most dramatic difference in diet-induced weight gain between Dab2 conditional knockout (CKO) and heterozygous (HET) mice was observed around 3–4 months (or 12 to 16 weeks) after starting on a high fat diet, when the Dab2 HET mice weighed approximately 30% more than the Dab2 null CKO mice. The disparity was still substantial at the end of the experiment at 28 weeks, at around 20% difference (Fig. 1a). The wild-type mice were indistinguishable in diet-induced weight gain from the *dab2* heterozygous mice, as shown at the 28-week time point (Fig. 1a). The differences between the *dab2* HET and CKO mice have no statistical significance when on a normal chow, but are significant (p < 0.005) when on a high fat diet.

Typically in the diet experiments, we placed young mice on a designated diet (either high fat or normal) once the pups were genotyped and weaned at 3–4 weeks of age. We also tested the impact of a high fat diet on older mice (6 months). In the experiments, the littermates were first kept on a normal chow over a 5-month period (following weaning), and were switched to a high fat diet for another 3 months (Fig. 1b). The body weight of mature mice of 6 or more months of age increased at a lower rate on normal chow, and accelerated somewhat when switched to a high fat diet. Surprisingly, we observed no difference in weight gain between the *dab2* conditional knockout and heterozygous mice when the high fat diet started at 6 month of age (Fig. 1b). In numerous tests in addition to the specifically designed experiments shown in Fig. 1a,b, we consistently observed the lean phenotype of the Dab2 null mice on a high fat diet starting at 3 weeks to three months of age, but no impact when high fat diet was started after 6 months of age. Thus, the effect of Dab2 on high fat diet-induced weight gain presents only in juvenile mice, and the impact is lost in mature/older mice. We conclude that Dab2 affects high fat diet-induced expeditious weight gain between 3 to 16 weeks of age.

Despite the striking lean phenotype of the Dab2 null mice fed a high fat diet, serum lipid profiles showed only subtle differences between the null (Sox2-cre mediated deletion) and heterozygous mice (Fig. 1c). Glucose and cholesterol levels had small yet statistically significant increases, though serum triglycerides were unchanged. Using PIXI-mus Small Animal DEXA densitometer to analyze tissue composition, we found the difference in fat mass accounted for the differential weight gain, while the lean mass was essentially the same between Dab2 null and heterozygous mice (Fig. 1d,e) (S-Fig. 1a,b). The adipose composition of the Dab2 HET mice was about double that of the CKO mice when on a high fat diet; the fat mass was 39% and 22% of body weight, respectively (Fig. 1e). The reduced fat deposition in the Dab2 null mice was very obvious upon dissection of the peritoneum (Fig. 1f) (S-Fig. 1a,b). White adipose tissues from all fat depots were reduced in Dab2 null mice: inguinal, 63%; subcutaneous, 76%; gonadal, 64%; and mesenteric, 71%, compared to those of heterozygous mice. These percentages were calculated from the weights determined (Fig. 1f). No change occurred in brown fat mass. The differences were statistically significant in all except brown fat, which showed no noticeable difference. In a controlled experiment, food intake was monitored by weighing the remaining chow daily, and no significant food intake was found between *dab2* HET and CKO mice (S-Fig. 1c). These data indicate that Dab2 deficiency leads to resistance to caloric-induced adipose tissue expansion and weight gain.

After being fed a high fat diet for 6 months, the lean *dab2* null mice exhibited a lower surge in blood glucose than the obese *dab2* heterozygous animals following a glucose challenge (Fig. 1g). Correspondingly, the lean *dab2* null mice also had a greater insulin response in lowering blood glucose than *dab2* heterozygous littermates in an insulin sensitivity test (Fig. 1h). Thus, *dab2* null mice appear to have normal glucose regulation even on a high fat diet, which causes an expanded adipocyte cell size but also a reduced cell number in the mutant animals. Therefore, an enlarged adipocyte size alone, without an increase in cell number and thus adipose masses, does not cause insulin resistance seen in obese mice with increased and enlarged adipocytes.

### Dab2 gene deletion affects expansion of adipose cell number but not cell size in mice

**Figure 2:**
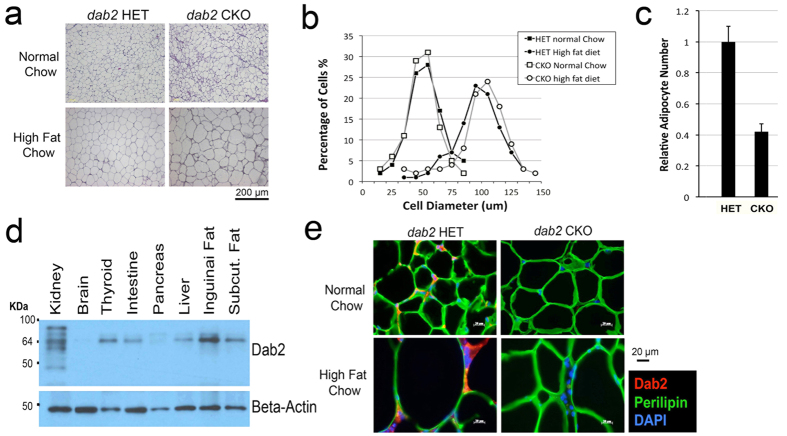
Impact of high fat diet on cell size and number of Dab2-deficient mice. (**a**) H&E sections of gonadal adipose tissues are shown for *dab2* CKO and HET controls fed either normal or high fat chow. (**b**) The images were analyzed using the MetaMorph Image Analysis Software for the diameter of the adipocytes as an indication of cell size. The cell size distribution is shown as percentage of cells at each increment of diameter. HFD induced a similar increase of adipocyte cell size in both *dab2* CKO and HET mice. (**c**) The relative number of adipocytes in gonadal adipose tissues from *dab2* CKO and HET mice on HFD was estimated based on the tissue mass and average cell diameter. (**d**) Dab2 protein expression was determined by Western blot in protein extracts from various tissues dissected from the mice. It was confirmed that Dab2 proteins were completely absent in all tissues from the *dab2* CKO mice. (**e**) Analysis by immunofluorescence microscopy of Dab2 and perilipin-1 in gonadal fat tissues from *dab2* CKO and HET mice fed with either normal or high fat diet.

Histological analyses showed that high fat diet increased the cell size of the adipocytes in both Dab2 null and heterozygous mice (Fig. 2a). Surprisingly, Dab2 null adipocytes were even slightly larger, rather than reduced size compared to those of wild-types (Fig. 2a), as confirmed by computer-assisted morphometric image analysis (Fig. 2b). Cell number of the adipose tissues was also estimated based on the fat mass and cell diameter: the adipocyte number in HET mice is 2.4-fold that of *dab2* CKO (Fig. 2c). Thus, we conclude that the absence of Dab2 in the mutant mice fed with a high fat diet does not significantly affect adipocyte cell size but rather affects the total cell number, and *dab2* CKO mice on high fat diet have an around 40% reduction in total fat cell number compared to HET mice.

Dab2 is abundant in the wild-type adipose tissues as shown by Western blot (Fig. 2d), and this was also confirmed by immunofluorescence microscopy analysis of gonadal adipose, where Dab2 staining was found as punctate dots in the narrow cytosolic space located between the perilipin-enclosed lipid droplets (Fig. 2e). From the public gene expression data sets, adipose tissue is shown to rank 3rd for the expression level of Dab2 among all mouse tissues and cell types analyzed, following bone marrow macrophages and kidney (S-Fig. 2), suggesting the possibility that Dab2 plays roles in adipocyte differentiation or function.

**Figure 3:**
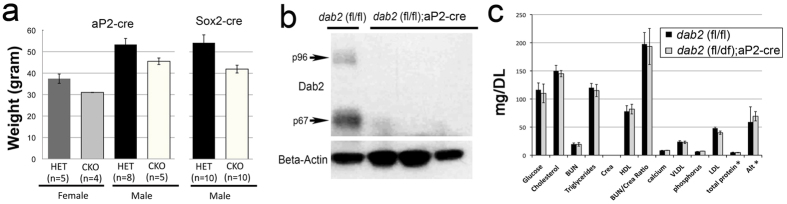
Impact of high fat diet on weight gain in mice of adipocyte lineage restricted *dab2* deletion. *dab2* (fl/fl) mice were crossed with aP2-cre mice to produce mice in which *dab2* gene was deleted only in adipocyte tissues (*dab2* (fl/df);aP2-cre) (adipocyte-restricted *dab2* knockout). (**a**) Both male and female adipocyte tissue-specific Dab2 deficient mice were challenged with a high fat diet starting at 7 weeks of age for an additional 28 weeks. The body weights were determined and compared with control littermates (*dab2* (fl/+);aP2-cre). Student’s T-test indicates that the weight difference between the two genotypes is statistically significant (p < 0.005). (**b**) Adipose tissues were collected from the *dab2* (fl/fl) and *dab2* (fl/df);aP2-Cre mice, and protein extracts were prepared from the tissues for Western blot analysis. The Western blot indicates that both Dab2 p96 and p67 isoforms were absent in adipose tissues collected from 3 *dab2* (fl/df);aP2-cre mice. (**c**) Blood chemistry analysis was performed on *dab2* (fl/df) and *dab2* (fl/df);aP2-cre mice that had been fed with an high fat diet but fasted for 12-hour before blood sampling. Results from 8 male mice in each group were averaged and standard errors are shown. The serum lipid and metabolite profiles are essentially identical between *dab2* (fl/df) and *dab2* (fl/df);aP2-cre mice. ALT, alanine aminotransferase.

We also examined whether the effect of Dab2 is adipose specific by testing in mice with *dab2* deletion restricted to adipose tissues using the aP2-cre (also known as Fabp4-cre) line^33^,^34^. By crossing the *dab2* (fl/fl) line with the aP2-cre line, we generated conditional knockout (*dab2* (fl/df);aP2-cre) and heterozygous control (*dab2* (fl/df)) mice. The adipose-restricted *dab2* conditional knockout mice also showed a reduced weight gain compared to heterozygous mice after being placed on a high fat diet (Fig. 3a). The average weight of female *dab2* conditional knockout (*dab2* (fl/df);aP2-cre) mice was 84% of control heterozygous females, and males were 87% of the HET mice, compared to 76% for the Dab2 null (*dab2 (*f/df);Sox2-cre) male mice (Fig. 3a). The efficiency of the aP2-cre for *dab2* deletion in adipose tissues was demonstrated for the absence of Dab2 by Western analysis of the adipose tissues (Fig. 3b). Serum lipid profiles were indistinguishable between mice with or without aP2-cre-mediated *dab2* deletion (Fig. 3c). Thus, *dab2* deletion within the adipocyte lineage affected high fat diet-induced weight gain, though the magnitude was not as profound as in mice with global *dab2* deletion using Sox2-cre.

### Adipocyte differentiation is deficient in Dab2 null cells through phosphorylation and nuclear exclusion of PPARγ

**Figure 4:**
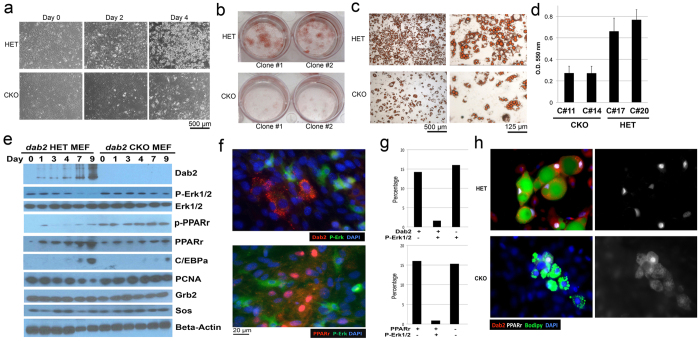
Increased MAPK activity and reduced capacity for adipose differentiation in Dab2-deficient embryonic fibroblasts and other adipocyte precursor cells. (**a**) *dab2* HET and CKO MEFs were used for differentiation into adipocytes following incubation for two days in “Induction Medium” and then culturing in “Differentiation Medium” for 4 to 14 days. Morphology of the cells is shown for Day 0, 2, and 4 in “Differentiation Medium”. (**b**) Following differentiation for 7 days in the “Differentiation Medium”, the cells were stained with Oil-Red O. Two independent clones each of Dab2-deficient (CKO) and -positive (HET) MEF cells are shown. (**c**) Representative magnified images of the cells stained with Oil-Red O are shown. (**d**) The amount of Oil-Red O was quantified spectrophotometrically at 550 nm, and triplicate assays from two independent clones are shown for each genotype. The differences between HET and CKO are statistically significant (p < 0.005). (**e**) Total cell lysates were prepared at each day during differentiation and analyzed by Western blot. All gels were derived from the same cell lysates and processed in an identical condition. (**f**) Representative examples of immunofluorescence staining for p-Erk1/2 and Dab2, and p-Erk1/2 and PPARγ are shown for the *dab2* HET MEFs at an early stage of differentiation (Day 2). (**g**) Quantitation indicates that p-Erk1/2 and Dab2 expression were inversely correlated, and the expression of p-Erk1/2 and PPARγ were also inversely correlated. About 200 cells from 5 images acquired for each sample were quantified to produce the statistics. (**h**) Representative examples of immunofluorescence microscopy detection of PPARγ, Dab2, and Bodipy are shown for the cells at a later stage (Day 4) of differentiation. A visual observation indicates that PPARγ expression and nuclear localization, Dab2 expression, Bodipy positivity in the same cell correlate in the Dab2 HET cell population. In Dab2 null cells, the majority of PPARγ staining is cytoplasmic rather than nuclear.

**Figure 5:**
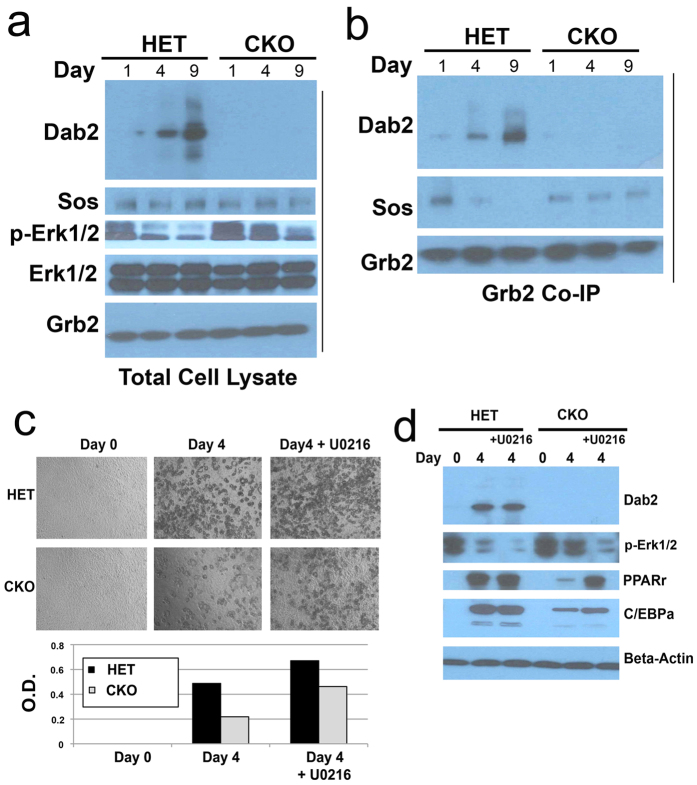
Restraint of Erk1/2 activation through modulation of Sos1-Grb2 association by induced Dab2 during adipocyte differentiation. *dab2* HET and CKO MEFs were subjected to adipose differentiation following the two-step protocol: 2 days in the “Induction Medium” followed by a time course in the “Differentiation Medium”. Grb2 and associated proteins were immunoprecipitated from Dab2 HET and CKO MEF cells on days 0, 1, 4, 9 of adipose differentiation. (**a**) Western blot analysis of Dab2, p-Erk1/2, Sos1, and Grb2 was performed on total cell lysates. (**b**) Western blot analysis of Dab2, Sos1, and Grb2 was performed on anti-Grb2 co-immunoprecipitation (co-IP) samples. (**c**) *dab2* HET and CKO MEFs were first treated with “Induction Medium” for 2 days and then were cultured in the “Differentiation Medium” to mature into adipocytes in the presence or absence of MEK inhibitor U0216 for 4 days. Morphology of the cells was recorded, the cells were stained with Oil-Red O, and the adipocyte content was quantified. (**d**) The total cell lysates were analyzed by Western blot for markers including Dab2, phospho-Erk1/2, PPARγ, C/EBPα, and beta-actin. All Western blots compiled within a panel were derived from the same cell lysates and processed in an identical condition.

**Figure 6:**
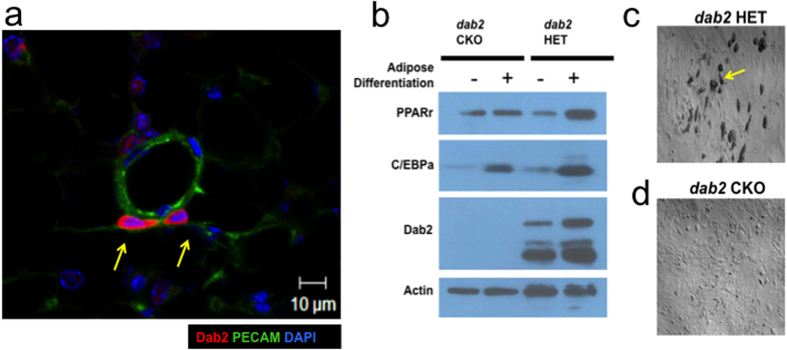
Adipose differentiation from cells of the stromal vascular fractions. Dab2-positive cells were identified to locate around blood vessels in adipose tissues. Cells of the stromal vascular fractions were isolated from inguinal adipose tissues of two-month-old *dab2* HET and CKO male mice. The cells were cultured for 1 week and then were subjected to differentiation for 2 days. (**a**) Inguinal adipose was sectioned and stained for PECAM (green, a marker for vesicular endothelial cells), Dab2 (red), and DAPI (blue). Dab2-positive cells (indicated by arrows) were found located around blood vessels in the adipose tissues. (**b**) Lysates were prepared from pre- and post-differentiation, and were analyzed by Western blot. All gels were derived from the same cell lysates and processed in an identical condition. (**c**) The morphology of the *dab2* HET cells are shown. The arrow indicates the presence of lipid droplets contained in pre-adipocytes of HET (but rare in CKO) cells.

The results that Dab2 is found expressed in adipocytes and that aP2-Cre lineage restricted *dab2* deletion also has a reduced weight gain suggest a cell autonomous requirement of Dab2 in adipocyte development. Thus, we investigated the effect of *dab2* deletion on adipocyte differentiation in cultured cells. Multiple mouse embryonic fibroblast (MEF) lines were prepared from embryos generated from crosses between male *dab2* (+/df);Sox2-cre and female *dab2* (fl/fl) mice, and the cells were genotyped and subjected to *in vitro* adipocyte differentiation. In all the 6 *dab2* CKO and 10 HET MEF lines assayed, a consistent reduction in the degree of adipose differentiation was observed in CKO compared to HET lines based on obvious morphological changes (Fig. 4a), and staining of adipocytes with Oil-Red O (Fig. 4b,c). Quantitation indicated that the lipogenic differentiation of the *dab2* CKO cells was reduced to about 40% of HET cells (Fig. 4d). Similarly, adipogenic markers, C/EBPα, Leptin, PPARγ1, and PPARγ2, were remarkably reduced in Dab2 CKO cells compared to HET MEFs, as of 43%, 58%, 53%, and 43% respectively (S-Fig. 3). In addition, we found that Dab2-deficient mesenchymal stem cells (S-Fig. 4) and embryonic stem (ES) cells (S-Fig. 5) also had a reduction in lipogenic differentiation compared to HET control cells in respective differentiation conditions. Multiple ES lines and mesenchymal stem cells preparations isolated from the mutant mice were tested and produced consistently similar results. Furthermore, Dab2 suppression using siRNA in the 3T3L1 adipocyte differentiation assay also reduced the adipogenic activity to 20–30% (S-Fig. 6). Thus, consistent and reproducible results generated from several cell types, and suppression of Dab2 by gene knockout or siRNA suppression, confirmed a cell autonomous role of Dab2 in promoting adipocyte differentiation.

We subsequently investigated the impact of Dab2 on signaling pathways during adipogenic differentiation of MEFs. In multiple experiments, we observed the robust induction of Dab2 expression as HET and wild-type cells progressively differentiated, accompanying an increasing PPARγ and C/EBPα expression (Fig. 4e). In comparison, the induction of PPARγ and C/EBPα was blunted in the *dab2* CKO cells. However, despite the reduced protein, PPARγ phosphorylation as detected by phospho-specific antibodies was elevated in Dab2-deleted cells. In HET (and wild-type) cells, Erk1/2 activation (phospho-Erk1/2 level) was reduced at later time points of differentiation, accompanying a greater induction of Dab2 expression (Fig. 4e). In Dab2-deficient cells, in contrast, the phospho-Erk1/2 level remained elevated especially at the later days, but total Erk1/2, Grb2, Sos1, and beta-actin were unaltered (Fig. 4e). Growth suppression accompanied adipocyte differentiation, indicated by the reduction in the proliferation indicator PCNA. However, PCNA reduction was not observed in *dab2* CKO cells (Fig. 4e).

On day 2 of differentiation when Dab2 expression was only slightly induced, a clear correlation between the presence of Dab2, phospho-Erk1/2, and PPARγ within an individual cell was observed by immunofluorescence microscopy (Fig. 4f). Of a designated cell, Dab2 and phospho-Erk1/2 showed a mutually exclusive expression pattern: when Dab2 was induced, phospho-Erk1/2 was negative, as quantified in over 400 cells examined. The cells were positive for Dab2 in 14%, positive for phospho-Erk1/2 in 16%, but only 1.6% of the cells were positive for both Dab2 and phospho-Erk1/2 (Fig. 4g). Similarly, nuclear PPARγ and phospho-Erk1/2 were also mutually exclusive (Fig. 4f), with only about 1% of the cells positive for both phospho-Erk1/2 and PPARγ (Fig. 4g). At later days (for an example, day 4), the differentiating adipocytes were identified using Bodipy staining of the intracellular lipid droplets and were also positive for Dab2 (Fig. 4h). In these Bodipy-positive cells, PPARγ showed strong positive nuclear staining. In comparison, Bodipy-positive cells were fewer and PPARγ appeared cytoplasmic in the *dab2* CKO cells (Fig. 4h). These results demonstrate a close association between Dab2 induction, phospho-Erk1/2 suppression, reduced PPARγ phosphorylation, and PPARγ nuclear localization. The data suggests that elevated Erk1/2 activation in *dab2* CKO cells leads to phosphorylation and nuclear exclusion of PPARγ, and consequently a reduction in adipogenic differentiation. The conclusion is also consistent in adipogenic differentiation of mesenchymal stem cells (S-Fig. 4), embryonic stem cells (S-Fig. 5), and 3T3L1 pre-adipocytes (S-Fig. 6).

### Induction of Dab2 during adipocyte differentiation causes uncoupling of Grb2-Sos1 complex and suppression of Erk1/2 activation

We considered a potential mechanism that Dab2 modulates the activity of the Ras-MAPK (Erk1/2) pathway by its binding to Grb2, thus competing with Sos1, a GTP exchange factor for Ras^23^,^24^,^25^. We first assessed the impact of Dab2 on the association of Grb2 and Sos1 in the process of adipocyte differentiation of MEFs using co-immunoprecipitation. In total cell lysates, induction of Dab2 in differentiating HET cells accompanied a reduction in phospho-Erk1/2, whereas Sos1, Grb2, and total Erk1/2 remained unchanged (Fig. 5a). In comparison, high phospho-Erk1/2 levels persisted in Dab2 null cells (Fig. 5a). Immunoprecipitation of Grb2 was used to determine its relative association with Dab2 or Sos1 (Fig. 5b). Western blot analysis of Dab2 and Sos1 in the immunoprecipitates indicated that on day 1 of differentiation, Sos1, but little Dab2, associated with Grb2 in HET cells. However, by days 4 and 9 of differentiation, Dab2 replaced Sos1 in the Grb2 immunoprecipitations (Fig. 5b). In the absence of Dab2, Sos1 remained at the same level in Grb2 immunoprecipitations prepared from the Dab2 null cells along the time course of differentiation (Fig. 5b). The results are consistent with the idea that Dab2 expression progressively increases during adipocyte differentiation, competes with Sos1 for binding to Grb2, consequently causes the dissociation of Sos1 from Grb2, and hence results in a reduced Ras activation and Erk1/2 phosphorylation/activation.

Since the above data suggest the main effect of Dab2 is to suppress Erk1/2 activation, we tested the impact of Erk1/2 inhibition on adipocyte differentiation in Dab2 null MEF cells. Inclusion of the MEK inhibitor U0216 for 4 days partially restored the degree of adipocyte differentiation of the *dab2* CKO cells (Fig. 5c). In the presence of the MEK inhibitor, differentiation of Dab2 null MEF cells reached the same level as that of HET cells, though the *dab2* HET cells achieved an even greater degree of adipocyte differentiation in the presence of the inhibitor (Fig. 5c). U0216 suppressed Erk1/2 phosphorylation and increased PPARγ levels in the Dab2 null cells (Fig. 5d). Thus, the results support the conclusion that Dab2 regulates adipocyte differentiation by suppressing Erk1/2 activation, which would otherwise phosphorylate and down regulate PPARγ^14^,^15^,^16^,^18^,^19^.

### Dab2 is essential for adipose differentiation of cells of the stromal vascular fractions

Since cells of the stromal vascular fractions (SVF) in adipose tissues were believed to be the endogenous sources of pre-adipocytes^35^,^36^,^37^, we investigated the impact of Dab2 on the ability of the SVF cells to undergo adipose differentiation. Particularly, pre-adipocytes are believed to be pericytes residing on the outer wall of blood vessels in the vascular network of adipose tissues^37^,^38^,^39^,^40^. We examined the blood vessels of adipose tissues for the expression of Dab2 in the potential pre-adipocyte population. Blood vessels in the adipose tissues were identified by staining with PECAM, a marker for vascular endothelial cells (Fig. 6a). Distinctive Dab2-positive cells were found surrounding the outer wall of blood vessels (Fig. 6a, arrows). From tabulations of multiple slides of adipose tissue samples, we estimated that around 20% of these pericytes, cells immediately surrounding the vascular structure, are Dab2-positive.

We further isolated and analyzed in cultures of the pre-adipocyte population from blood vessels within adipose tissues. These SVF cells were isolated from inguinal adipose tissues of young (8 week old) *dab2* HET and CKO male littermates, and were tested for adipose differentiation in cultures (Fig. 6b–d). Following 2 days in the differentiation medium, a significant number (around 12%) of the *dab2* HET stromal vascular cells showed adipogenic potential and exhibited apparent morphology of intracellular lipid droplets (Fig. 6c, arrow). In comparison, no or rare (less than 1%) lipid droplet-containing cells were found in the *dab2* CKO SVF cells (Fig. 6d). Differentiation was accompanied by an induction of PPARγ in HET but not in CKO cells, and CEBPα was induced to a lesser extend in the CKO cells (Fig. 6b). We also observed that, Dab2 protein was already present in SVF cells and only slightly induced following differentiation (Fig. 6b). The result is consistent with the idea that Dab2 is required for adipose differentiation of a pre-adipocyte stem cell population, likely the Dab2-positive pericytes, following exposure to excessive caloric intake.

### Discussion

The current investigation of a striking obesity resistant phenotype of Dab2 null mice revealed the mechanism of the Ras/MAPK pathway regulation in the differentiation and cell numerical expansion of adipocytes in mice. The Ras/MAPK pathway is activated by receptor tyrosine kinase through recruitment of Ras activator Sos1 through adaptor Grb2^41^,^42^. The association between Sos1 and Grb2 is regulated as a feed back loop^43^,^44^, and Dab2 competes with Sos1 for binding to Grb2^23^,^24^,^25^. The transfer of Grb2 from Sos1 to Dab2 is thought to occur at the endosomes, coinciding with the rearrangement of membrane proteins in the disassembly of adaptin and clathrin coats^25^. Thus, Dab2 suppresses Ras/MAPK signaling from the endomembranes^25^,^45^,^46^,^47^. Here we show that Dab2 regulation of Grb2/Sos1 association and thus signaling plays an essential role in suppressing the pathway in adipocyte differentiation in intact animals. The data demonstrate the induction of Dab2 during adipocyte differentiation, and that Dab2 promotes Grb2/Sos1 disassembly and reduces Ras/MAPK activation, which in turn modulates PPARγ expression, phosphorylation, and nuclear translocation. Thus, these data indicate that Dab2 induction and consequently Ras/MAPK suppression is a critical switch in modulating PPARγ activity and adipocyte differentiation. In cells of the SVF, Dab2 protein appears to be present and is positive in pericytes, the presumptive pre-adipocytes. Thus, Dab2 is required for adipose differentiation of this SVF pre-adipocyte population.

The study of Dab2 null mice also revealed several interesting clues about the development of adipose in mammals. First, the regulation of adipocyte cell number has a dominating role in controlling obesity in juvenile animals, since the lean Dab2 null mice have fewer adipocytes despite a greatly increased cell size. Second, the impact of Dab2 on diet-induced weight gain and adipose development was observed only in young mice, and older Dab2 null mice did not differ from control mice in response to the diet. These Dab2-positive pre-adipocytes are likely pericytes locating in the vascular structure of the adipose tissues. The observation implicates that a Dab2-dependent adipocyte precursor population exists in younger mice that can contribute to cell number expansion upon challenge with a high fat diet. This fat stem cell population is then diminished as the mice age, and older mice can regulate adipose tissue mass by mainly expansion of cell size rather than number. Lastly, expression of Dab2 and its subsequent action on suppressing Ras/MAPK activity is a pathway independent of PPARγ expression, and together the dual pathways determine the extent of adipogenic differentiation.

The high fat diet resistant phenotype of the Dab2 CKO mice is very similar to that of the Erk1 knockout mice^12^, though the two genetic mutations should have opposite impact on the pathway. Based on several studies of the Ras/MAPK pathway on adipogenesis, it is proposed that Erk1, but not Erk2, is essential for the recruitment and development of a pre-adipocyte population^13^. However, both Erk1 and Erk2 are suppressive, at a later time, for the adipose differentiation of the pre-adipocytes. Thus the current results are supportive of the conclusion from Bost *et al*.^13^, that Dab2 is essential for the differentiation of the pre-adipocyte population, but not the recruitment and development of the progenitor cells that is dependent on Erk1, by suppressing the activity of Erk1/2.

The current results allow us to suggest a model in which Dab2 modulates the Ras/MAPK pathway and thus impacts the differentiation and recruitment of new adipocytes into adipose tissues. According to this model, excessive calories induce the expression of pro-adipogenic genes such as PPARγ. Normally, Ras/Erk1/2 activity suppresses PPARγ and thus lipogenic differentiation. The presence of Dab2 leads to the suppression of Ras/Erk1/2 activity through Dab2 binding and sequestration of Grb2 from the Grb2/Sos complex, which results in the reduction of Ras/Erk1/2 activity. Consequently, PPARγ enters the nucleus to initiate a transcription program that promotes adipogenic differentiation.

Classical studies have suggested that adipocyte number is relatively constant despite loss or gain weight for humans^48^,^49^. Feeding studies also indicate juvenile animals are more subjective to high caloric induction of adipose numerical expansion^50^,^51^,^52^,^53^. Thus, intervention at younger age may be a critical period for countering obesity^54^. Existence of a pre-adipocyte population has been established and characterized^55^,^56^,^57^. High fat diet-induced adipocyte differentiation has been recently demonstrated^57^,^58^,^59^. However, our current finding suggests that there is a sharp age-dependent capability of the animals to induce an expansion of adipocyte number, and the commitment of the pre-adipocyte population is mediated by the induction of Dab2.

Future studies may determine if Dab2 regulation of diet-induced adipocyte expansion found in mouse models is applicable to human adolescents, and if the mechanism can be explored to counter childhood obesity.

## Methods

### Breeding, genotyping, and use of mouse models

We produced and used a *dab2* flox (fl) mouse line constructed to delete both exons 3 and 4 of the *dab2* gene, which leads to a null allele^20^. Here, we use “*dab2*” for the mouse gene, “Dab2” for the protein, and “DAB2” refers to the human gene. From our previous study, the *dab2* (fl/fl) mice were found to be indistinguishable from the wild-type mice in the absence of Cre recombinase. To delete the *dab2* gene conditionally, female *dab2* (fl/fl) mice were crossed with male *dab2* (+/df);Sox2-cre or *dab2* (+/df);aP2-cre to produce progenies for experiments. Litters with the *dab2* (fl/df):Sox2-cre genotype were designated as conditional knockouts (CKO), while *dab2* heterozygous (HET), *dab2* (+/df):Sox2-cre mice were used as controls. Heterozygous *dab2* mice from the same litters showed no detectable phenotypes and were used as controls for the conditional knockout mice. The genetic background of these mice was predominately C57BL/6 following seven rounds of back crossing, and likely only a minor 129/Sv contribution (originating from gene targeting in ES cells) persisted.

Sox2-cre mice (Tg(Sox2-cre)#Amc/J) (29) and aP2/Fabp4-cre mice (B6.Cg-Tg(Fabp4-cre)1Rev/J)^33^ were purchased from Jackson Laboratories. The mouse colonies were housed inside the barrier area of the mouse facility of University of Miami Miller School of Medicine, and PCR genotyping was performed as described previously^20^.

Mice were routinely reared on a regular enriched diet, 5K20 chow (10% fat, from LabDiets, Inc.). D12492 chow (Research Diets, Inc.) that contains 60% kcal from fat was used as a high fat diet (HFD) regimen for inducing weight gain/obesity, and D12450B (20% protein, 70% carbohydrate, and 10% fat) was used as normal control diet. Animals were housed on a 12 hr:12 hr light:dark cycle with *ad libitum* access to food and water. Body weight was measured weekly using a lab balance.

All experiments using mice were performed in accordance with NIH guidelines and all protocols were reviewed and approved by IACUC of the University of Miami Miller School of Medicine.

### Quantification of tissue composition

The fat and lean tissues of Dab2 conditional knockout (CKO) and heterozygous (HET) littermates were determined non-invasively using the PIXImus small animal dual-X-ray absorptiometry (DEXA) system (PIXImus™, GE Medical Systems, Fitchburg, WI: http://piximus.com/). Prior to analysis, the mice were anesthetized with an intraperitoneal injection of ketamine/xylazine cocktail, and placed in the prone position such that the subcranial tissues could be scanned by the Lunar PIXImus Densitometer.

### Profiling of blood lipids and metabolites

Mice were fasted for 4–6 hours and then anesthetized prior to tissue collection. Blood was collected by cardiac puncture, and the tissues and fats to be examined were dissected and collected. To obtain serum, blood samples were allowed to clot at room temperature for 15 min, and the serum was collected following centrifugation for 10 min at maximum speed in a bench top clinical centrifuge, and stored at −80 °C until use. Lipid chemistry analysis was performed using a Vitros 250 chemistry analyzer (Johnson & Johnson) by the University of Miami Comparative Pathology Laboratory Core. The levels of serum cholesterol, triglyceride, and high-density lipoprotein (HDL) were determined colorimetrically following reactions. Low-density lipoprotein (LDL) and very low-density lipoprotein (VLDL) levels were estimated as follows: VLDL = 1/5 of triglycerides; LDL = total cholesterol minus HDL and VLDL.

### Preparation of primary cells

Primary mouse embryonic fibroblasts (MEF) were isolated from E12.5 embryos generated from crosses of between *dab2* (fl/fl) females and *dab2* (+/df):Sox2-cre males (Males were used to avoid transmission of Cre protein from oocytes). Briefly, the head of dissected embryos was first removed and used for PCR genotyping and the remainder was minced using a scalpel. After incubation for 10 min in 0.25% trypsin at 37 °C, the mixture was pelleted by a brief centrifugation and plated in a T75 flask in DMEM media supplemented with 10% FBS. The cells from the outgrowth were harvested and expanded. Western blot analysis of the cells was used to confirm the genotype obtained from PCR of the head tissues.

Embryonic stem (ES) cells of Dab2 positive and null were generated from E3.5 mouse blastocysts/embryos following crosses of *dab2* (df/+) parents as previously described^20^.

Mesenchymal stem cells (MSCs) were isolated from bone marrow and cultured as described^60^. Briefly, cells were aspirated from bone marrow harvested from tibia and femur of control and mutant mice and cultured in DMEM containing 15% FBS. After incubation overnight, non-adhesive cells were removed and discarded. After 2–3 passages, the remaining cells were MSCs and were used for differentiation through the standard protocol^10^.

Stromal vascular cells (SVCs) containing pre-adipocytes were isolated from adipose tissues according to published methods^61^,^62^. Briefly, inguinal white adipose tissues were dissected from mice and sliced to small fragments with scalpel. The minced adipose tissues were transferred to a 15 ml Falcon tube, digested with 2 mg/ml Collagenase Type IV (Sigma C-5138) in DMEM/F12 with shaking (200 rpm) at 37 °C for 3 hours. After incubation, the mixtures were filtered through a cell strainer with 40 μm pore size and centrifuged at 500 g for 10 minutes to collect the SVCs. Around one million cells were plated on gelatin-coated coverslip, placed in 6-well plate tissue culture dish with culture medium. After culturing for 2 days, the cells were stained with PPARγ, C/EBPα, and BODIPY for the analysis of adipocyte precursor cells.

### Adipocyte differentiation assay and Oil-Red O staining

Mutant and wild-type MEFs were used for assays of adipocyte differentiation in culture. Monolayer cultures at about 50% confluency were first treated for 2 days with the “Induction Medium” (DMEM supplemented with 10% FCS, 8 μg/ml biotin, 4 μg/ml pantothenate, 0.5 mM 3-isobutyl-1-methylxanthine (IBMX), 1 μM dexamethasone, 10 μg/ml insulin (Sigma-Aldrich), and 10 μM troglitazone. The induced cells were than cultured in “Differentiation Medium” (DMEM supplemented with 10% FCS, 8 μg/ml biotin, 4 μg/ml pantothenate, and 10 μg/ml insulin) for up to 12 days to achieve differentiation to adipocytes. All the compounds were purchased from Sigma-Aldrich if not specified.

Bone marrow-derived primary MSCs were used for adipocyte differentiation following the standard protocol^10^. Briefly, the cells at 2–3 passages in culture were differentiated into adipocyte lineage by incubation with 5 *μ*g/ml insulin, 1 *μ*M dexamethasone, and 5 *μ*M IBMX for 2 days. The cells were then maintained in 10% FBS with 5 *μ*g/ml insulin for 7 days to complete differentiation.

For adipocyte differentiation from ES cells, a published protocol was followed (10). Multiple clones of Dab2 null and positive control pluripotent ES cells were prepared by culturing for two days to remove residual MEF feeder cells^20^. The ES cells were then maintained in suspension cultures without LIF but in the presence of 1 μM retinoic acid (RA) for 7 days to form embryoid bodies. The aggregated cells were then plated on cell culture dishes to allow adhesion and spreading, and were subsequently differentiated into adipocytes according to the standard protocol using the two-step procedure. Briefly, the adherent cells derived from embryoid bodies were primed by incubation with 5 *μ*g/ml insulin, 1 *μ*M dexamethasone, and 5 *μ*M IBMX for 2 days. After this induction, cells were maintained in 10% FBS with 5 *μ*g/ml insulin for 7 days to fully differentiate into adipocytes.

To inhibit Ras/MAPK pathway during adipocyte differentiation assays, the MEK inhibitor U0126 (Sigma-Aldrich) in DMSO was added to the cultures to a final concentration of 1 μM. It appeared that the inhibitor did not show significant cytotoxicity during the 4-day incubation.

Oil-Red O staining was performed to visualize the intracellular lipid droplets. Briefly, 0.5 g of Oil-Red O was dissolved in 100 ml isopropanol overnight with agitation. After sedimentation, the Oil-Red O solution and water were mixed in a 6:4 ratio to making the working solution. The differentiated adipocytes were fixed in 4% PFA for 1 hour after washing 3 times with PBS, air dried, and incubated for 15 min with freshly filtered Oil-Red O working solution. After removing the Oil-Red O solution and washing 3 times with PBS, the cells were imaged and quantitated using bright field microscopy. The Oil-Red O dye associated with the adipocytes was extracted with 100% isopropanol and the absorbance was measured at 550 nm.

### Adipocyte cell size measurement

The cell size/diameter of adipocytes was measured from histological slides stained with H&E using the MetaMorph Image Analysis Software. The red channel color of the original RGB images was selected and converted to black and white picture, which was used for histomorphometric quantification by the computer program. The individual cell size estimated was imported into a spreadsheet format and subjected to further calculation and analysis.

### Co-immunoprecipitation and Western blots

Cells in a 6-well plate were lysed with 0.5 ml of cold NP-40 IP buffer (1% NP-40, 150 mM NaCl, 50 mM Tris-HCl, 10 mM EDTA, pH 8.0) containing protease and phosphatase inhibitors (Pierce Biotech). The nuclear fraction was removed by centrifuging at 14,000 rpm for 20 min at 4 °C. The supernatant was collected and incubated with 10 μl of anti-Grb2 antibodies for 3 hours at 4 °C, and immunoprecipitation was performed using Dynabeads protein G immunoprecipitation kit according to the manufacturer’s protocol (Invitrogen). The beads were collected by the magnet device and washed three times with IP buffer. Collected antibodies with bound proteins were eluted by boiling in SDS-sample buffer, and then the protein extracts were used for Western blot analysis.

Primary antibodies and their dilution used include: anti-Dab2 (1/5,000) (BD Transduction Laboratories, 610465), anti-Beta-actin (1/5,000) (BD Transduction Laboratories, 612657), anti-Sos1 (1/1,000) (BD Transduction Laboratories, 610096), anti-Grb2 (1/2,000) (BD Transduction Laboratories, 610112 and Santa Cruz, sc-255), anti-Erk1/2 (1/5,000) (BD Transduction Laboratories, 610408), anti-phosphoErk1/2 (1/1,000) (Cell Signaling), anti-PCNA (1/2,000) (Santa Cruz, sc-56), anti-Lamin A/C (1/2,000) (BD Transduction Laboratories, 612162), anti-C/EBPα (1/1,000,) (Santa Cruz, sc-61), and anti-PPARγ (1/1,000) (Santa Cruz, H-100).

For Western blotting, the secondary antibodies were conjugated with horseradish peroxidase (HRP) and were used (1/5,000 dilution) following the instructions from the manufacturer (rabbit and mouse secondary antibodies from BioRad; goat secondary antibodies from Jackson ImmunoResearch). SuperSignal West Extended Duration Substrate (Pierce Biotech) was used for chemoluminescence detection of specific proteins.

### Histology, immunofluorescence, and confocal microscopy

Tissues were fixed with formalin, embedded in paraffin, and sectioned with a microtome at 6 μm thickness. Slides were dewaxed in a graded ethanol series, washed in water and boiled in antigen retrieval solution (10 mM sodium citrate, pH 6.0) prior to addition of antibodies.

Primary antibodies used were: anti-PPARγ, anti-C/EBPa, anti-Dab2, and anti-phospho-Erk1/2. Species-specific secondary antibodies were conjugated with the appropriate Alexa fluorochrome for simultaneous imaging of multiple antigens. DAPI (4′-6-diamidino-2-phenylindole) was used as a generic counterstain and applied in the terminal washing stages of the procedure. For lipid droplet staining, BODIPY 495/503 (fluorescent dye based on dipyrrometheneboron difluoride core structure) (Invitrogen) was diluted at 1:1,000 and added at the same time as secondary antibodies. In detection of multiple antigens with fluorescence microscopy, Bodipy was imaged last to avoid the persistent fluorescence upon stimulation of the dye. Confocal imaging was performed with an inverted Zeiss LSM510/uv Axiovert 200M laser scanning confocal microscope operated by Zeiss LSM software. Images were acquired with three sequential scan tracks. Objectives used included Plan-Apochromat (x63, 1.4 N/A) and Plan-Neofluar (x40, 1.3 N/A).

For immunohistochemistry, the secondary antibodies were HRP-conjugated (Vector Laboratories, CA, USA) and were detected by a DAB Peroxidase Substrate Kit (Vector Laboratories, CA, USA) followed by a hematoxylin counterstain.

### Quantitative RT-PCR for the analysis of gene expression

Total RNA was harvested from MEF cells using the Qiagen RNAeasy kit. cDNAs were prepared from the extracted RNA by reverse transcription with iScript™ cDNA Synthesis Kit according to the manufacturer’s instructions. Quantitative RT-PCR was performed using CFX Connect™ Real-Time PCR Detection System with 2X SYBR^®^ Green Supermix and specific primers. The primers used were: ***GAPDH***: 5′ AGT GGA GAT TGT TGC CAT CAA CGA CC; and 3′ GGA CTG TGG TCA TGA GCC CTT CC; ***PPARγ1***: 5′ TGA AAG AAG CGG TGA ACC ACT G, and 3′ TGG CAT CTC TGT GTC CAA CCA TG; ***PPARγ2***: 5′ GTT TTA TGC TGT TAT GGG TG, and 3′ GTA ATT TCT TGT GAA GTG CTC ATA G; ***C/EBPα***: 5′ GCC ATG GCC TTG ACC AAG GAG, and 3′ GAA CAG CAA CGA GTA CCG GGT A; ***leptin***: 5′ GGG ATG GCT CTT ATC TCT ACT TGC T, and 3′ CAC CAG GCT CCC AAG AAT CAT GTA.

## Additional Information

**How to cite this article**: Tao, W. *et al*. Disabled-2 Determines Commitment of a Pre-adipocyte Population in Juvenile Mice. *Sci. Rep.* **6**, 35947; doi: 10.1038/srep35947 (2016).

## Supplementary Material

Supplementary Information
