# Supplementary material for: Disabled-2 Determines Commitment of a Pre-adipocyte Population in Juvenile Mice
Source: Sci Rep. 2016 Oct 25;6:35947. doi: 10.1038/srep35947 (PMC5078790; doi:10.1038/srep35947)
Supplement: Supplementary Information [file srep35947-s1.pdf]

Supplementary Materials

Title: **Disabled-2 Determines Commitment of A Pre-adipocyte Population in Juvenile Mice**

Wensi Tao[1,2], Robert Moore[1], Yue Meng[1,2], Toni Yeasky[1], Elizabeth R. Smith[1], and Xiang-Xi Xu[1,2,+]

[1]Department of Cell Biology; [2]Graduate Program in Molecular Cell and Developmental Biology, Sylvester Comprehensive Cancer Center, University of Miami Miller School of Medicine, Miami, FL 33136 USA

Supplementary Figures 1to 6 and Legends

Sup-Figure 1

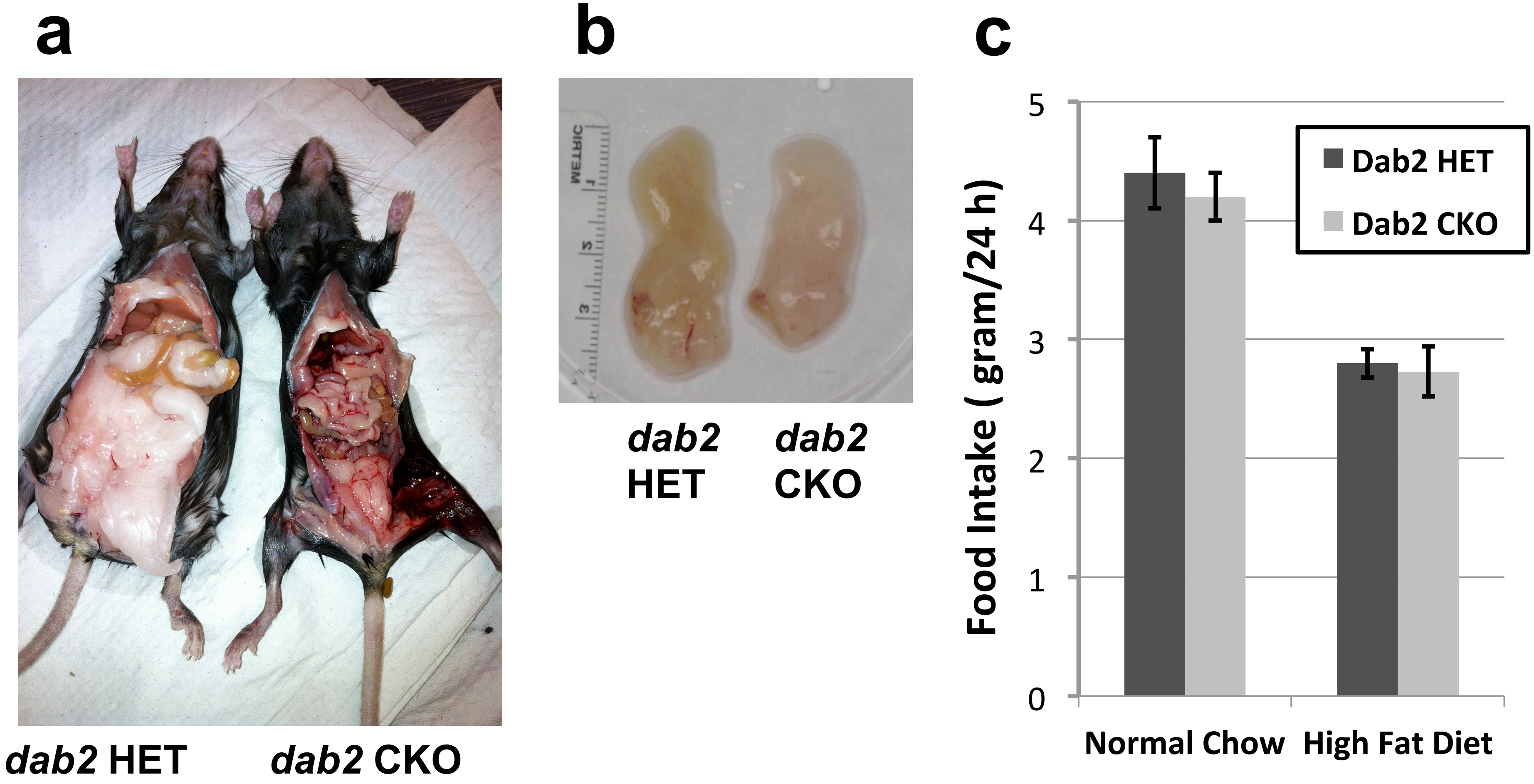
a
b
c
dab2 HET
dab2 CKO
dab2
HET
dab2
CKO
Food Intake (gram/24 h)
0
1
2
3
4
5
Dab2 HET
Dab2 CKO
Normal Chow
High Fat Diet

Sup-Figure 2

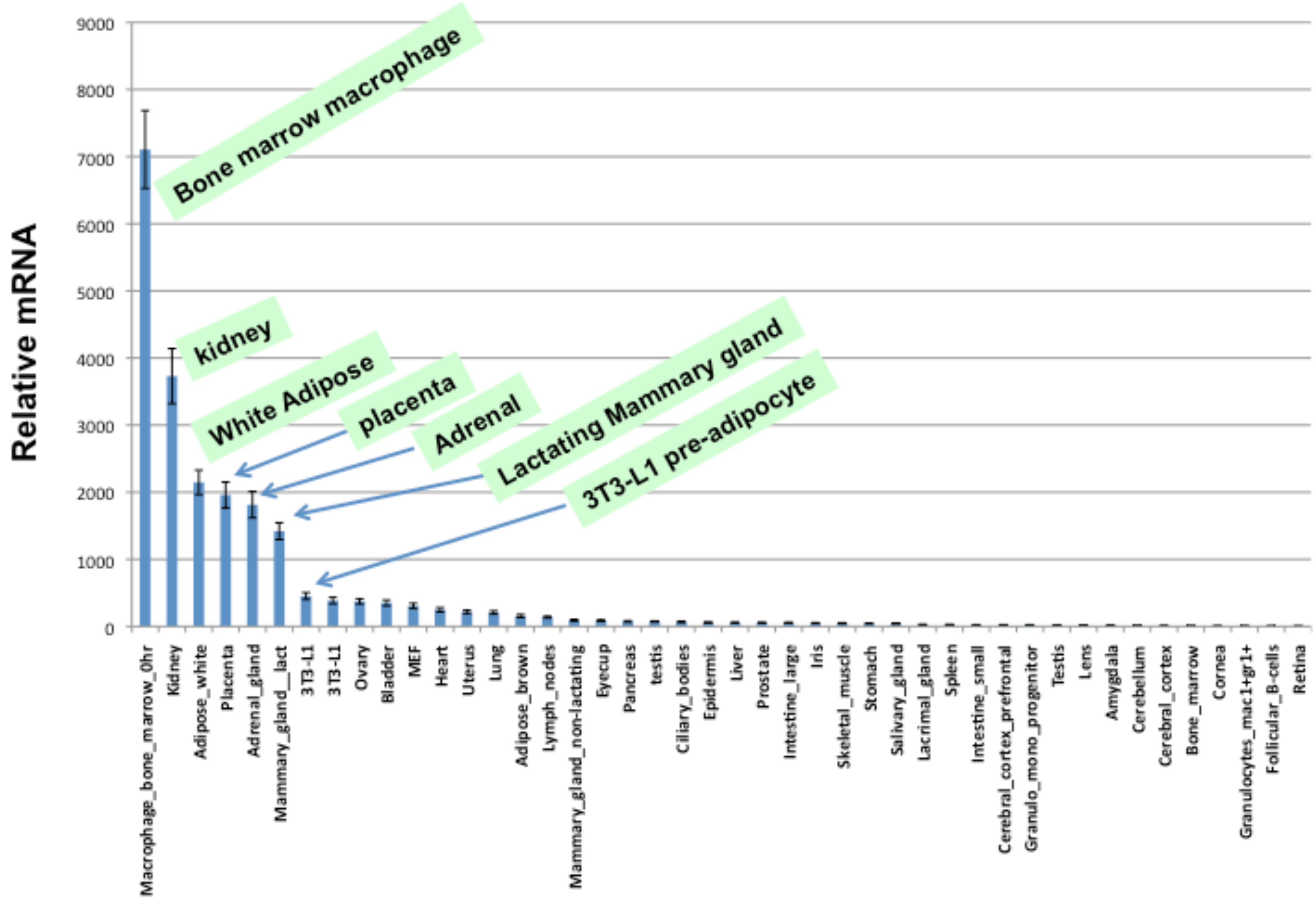

Relative mRNA
9000
8000
7000
6000
5000
4000
3000
2000
1000
0
Bone marrow macrophage
kidney
White Adipose
placenta
Adrenal
Lactating Mammary gland
3T3-L1 pre-adipocyte
Macrophage_bone_marrow_0hr
Kidney
Adipose_white
Placenta
Adrenal_gland
Mammary_gland__lact
3T3-L1
3T3-L1
Ovary
Bladder
MEF
Heart
Uterus
Lung
Adipose_brown
Lymph_nodes
Mammary_gland_non-lactating
Eyecup
Pancreas
testis
Ciliary_bodies
Epidermis
Liver
Prostate
Intestine_large
Iris
Skeletal_muscle
Stomach
Salivary_gland
Lacrimal_gland
Spleen
Intestine_small
Cerebral_cortex_prefrontal
Granulo_mono_progenitor
Testis
Lens
Amygdala
Cerebellum
Cerebral_cortex
Bone_marrow
Cornea
Granulocytes_mac1+gr1+
Follicular_B-cells
Retina

Sup-Figure 3

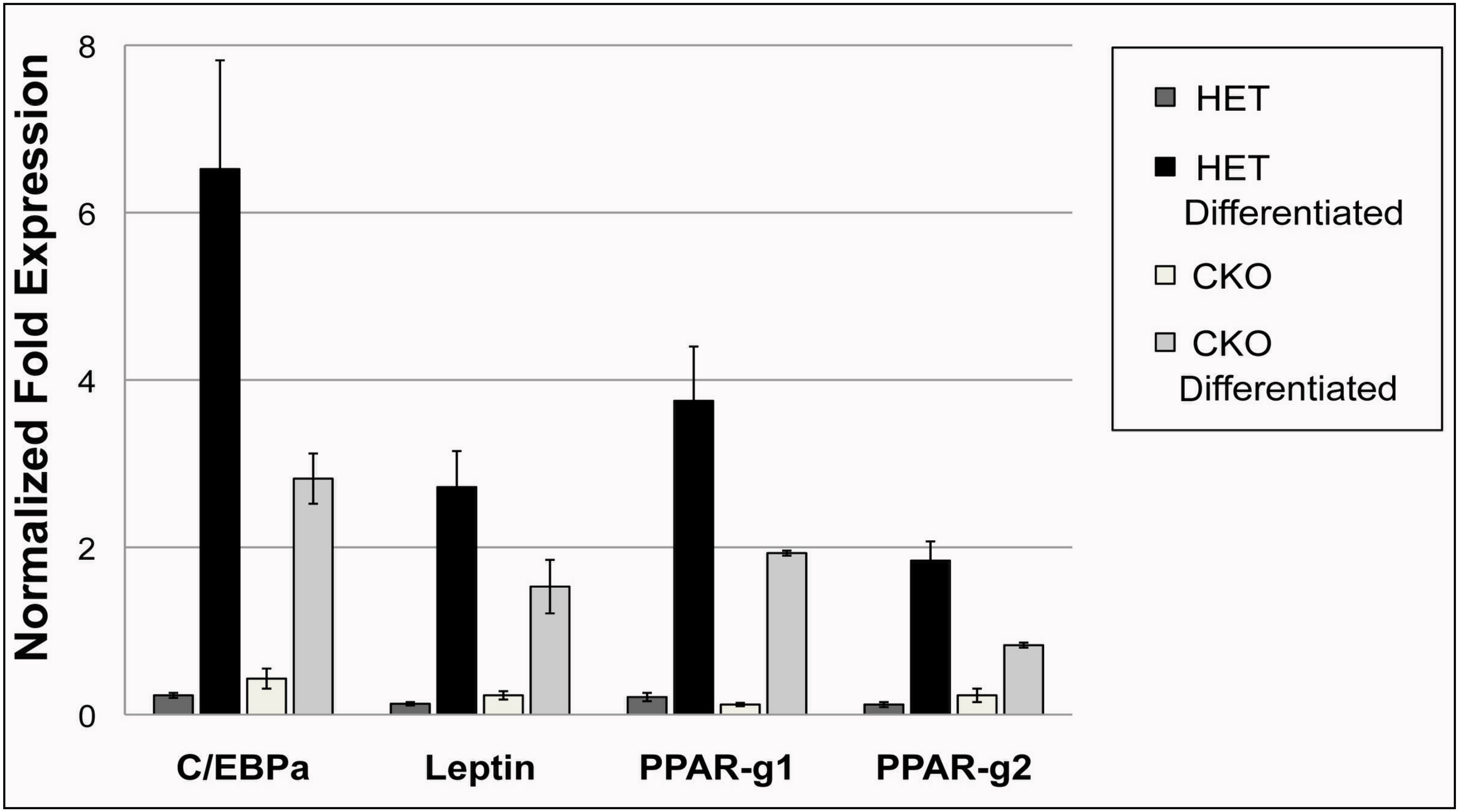

Normalized Fold Expression
8
6
4
2
0
C/EBPa
Leptin
PPAR-g1
PPAR-g2
HET
HET Differentiated
CKO
CKO Differentiated

Sup-Figure 4

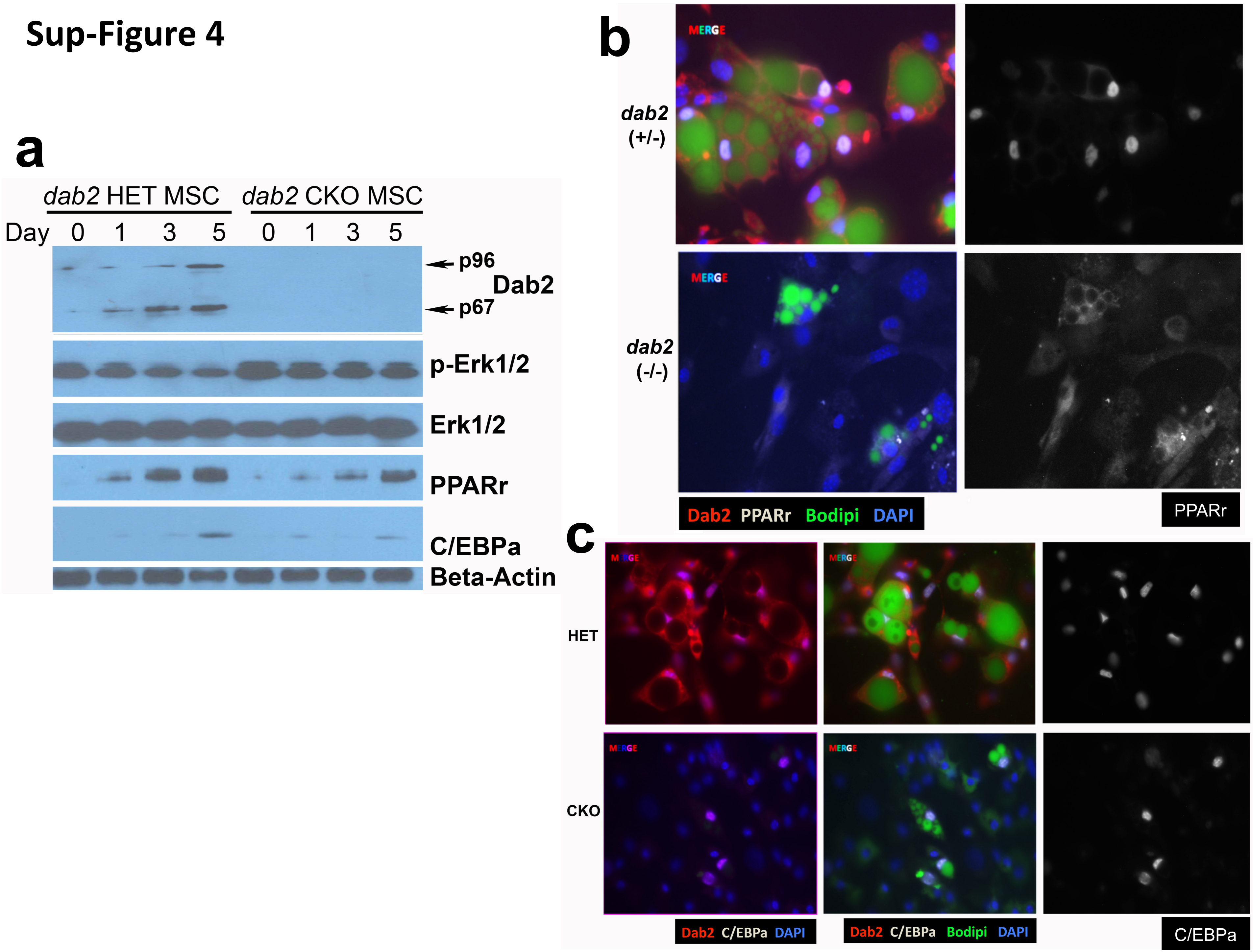
a
dab2 HET MSC
dab2 CKO MSC
Day 0 1 3 5 0 1 3 5
p96
Dab2
p67
p-Erk1/2
Erk1/2
PPARr
C/EBPa
Beta-Actin
b
dab2 (+/-)
dab2 (-/-)
MERGE
Dab2 PPARr Bodipi DAPI
PPARr
c
HET
CKO
MERGE
Dab2 C/EBPa DAPI
Dab2 C/EBPa Bodipi DAPI
C/EBPa

Sup-Figure 5

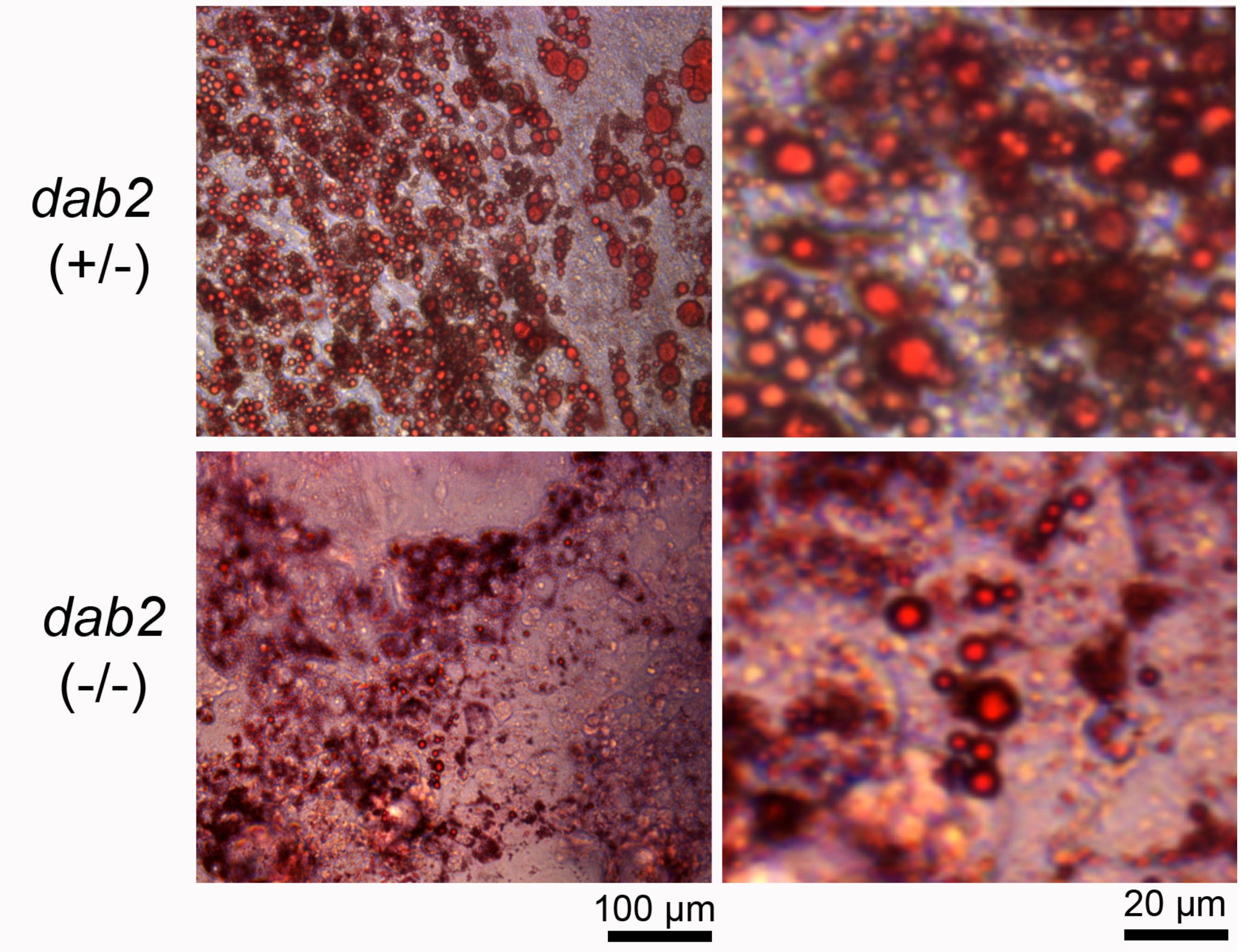

Sup-Figure 6

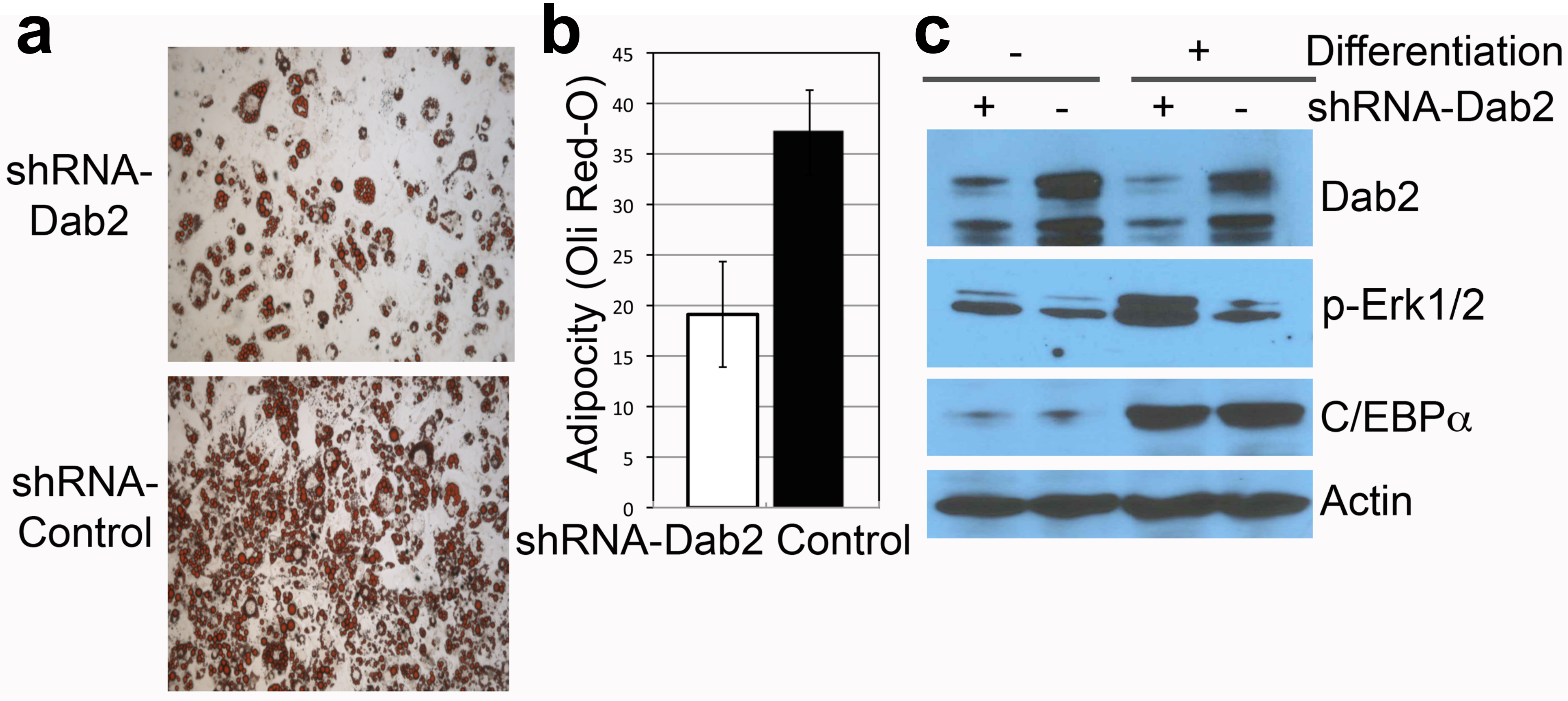
a
shRNA-Dab2
shRNA-Control
b
Adipocity (Oli Red-O)
45
40
35
30
25
20
15
10
5
0
shRNA-Dab2
Control
c
-
+
Differentiation
+
-
+
-
shRNA-Dab2
Dab2
p-Erk1/2
C/EBPα
Actin

6-19-2016

Supplementary Materials

Title: **Disabled-2 Determines Commitment of A Pre-adipocyte Population in Juvenile Mice**

Wensi Tao[1,2], Robert Moore[1], Yue Meng[1,2], Toni Yeasky[1], Elizabeth R. Smith[1], and Xiang-Xi Xu[1,2,+]
[1]Department of Cell Biology; [2]Graduate Program in Molecular Cell and Developmental Biology, Sylvester Comprehensive Cancer Center, University of Miami Miller School of Medicine, Miami, FL 33136 USA

Supplementary Figures, Legends

Supplemental Figure 1. Dab2 null mice were resistant to high caloric diet-induced obesity. (**a**) Representative examples of the exposed peritoneal fat tissues comparing a *dab2* CKO and a HET littermate after being placed on a HFD for 6 months. (**b**) Representative examples of dissected gonadal fats comparing a *dab2* CKO and a HET littermate after being placed on a HFD for 6 months. Excessive accumulation of fat tissues is apparent in the Dab2 HET mouse. In comparison, fat tissues are not remarkable and smaller in size in the Dab2 null mice (CKO). (**c**) Monitoring of food intakes in the groups of mice tested and showed in Figure 1A. The groups (10-12 mice per group, housed 3-4 mice per cage) of Dab2 CKO and HET male mice at 7 weeks of age were placed on either normal chow (NC) or high fat diet (HFD) for an additional 28 weeks. Near the end of the feeding period, food intakes were monitored for a week by weighting before and after, and the results were calculated to represent the gram of food consumed per animal per 24 hour. The data indicates that near the end of the feeding, mice on normal chow consumed more food than mice on high fat diet, and there were no significant differences between *dab2* HET and CKO mice in food intakes of either normal or high fat diet.

Supplemental Figure 2. Dab2 expression profile in mouse tissues. Microarray data sets were acquired from online public dataset GeneAtlas MOE430, gcrma (http://biogps.org/#goto=genereport&id=13132). The high-throughput gene expression profiling was generated from a diverse array of normal tissues, organs, and cell lines from mice. The final read outs were averages of four different probe sets, normalized to housekeeping genes,

and ranked according to the abundance of Dab2 mRNA. It is noted that white adipose tissues rank 3rd (followed bone marrow macrophages and kidney) for Dab2 mRNA abundance in all 45 tissues and cell types analyzed.

Supplemental Figure 3. Adipocyte marker expression in *dab2* HET and CKO MEFs following adipogenic differentiation. Quantitative RT-PCR analysis of mRNA from total RNA extracted from the MEFs before and after differentiation showed that the induced expression of adipogenic markers was remarkably reduced in Dab2 CKO cells compared to HET MEFs: C/EBPα, 43%; Leptin, 58%; PPARγ1, 53%; and PPARγ2, 43%. The differences in the expression all genes are statistically significant ($p < 0.005$) between HET and CKO cells.

Supplemental Figure 4. Adipocyte differentiation of Dab2-deficient mouse mesenchymal stem cells. Mesenchymal stem cells (MSCs) were prepared from bone marrow of *dab2* HET and CKO mice. These primary MSCs were used for differentiation into adipocytes following incubation for two days in "Induction Medium" and then culturing in "Differentiation Medium" for 4 to 9 days. (**a**) Induction of Dab2 expression and suppression of Erk1/2 activation during adipogenic differentiation of MSCs. Cell lysates were prepared for Western blot from the cells during the time course of differentiation. p-Erk1/2 levels decreased in *dab2* HET cells as differentiation progressed, but remained high in Dab2 CKO cells. (**b**) Representative examples of images from immunofluorescence microscopy of PPARγ, Dab2, and Bodipy are shown for the cells at Day 4 of differentiation. In the Dab2 HET cell population, PPARγ protein is nuclear, and the PPARγ-positive cells coincide with strong Dab2 expression and positive Bodipy staining. In Dab2 null cells, however, few cells contain Bodipy and the majority of detectable PPARγ is cytoplasmic rather than nuclear. (**c**) Representative examples of immunofluorescence microscopic images of C/EBPα and Dab2 are shown for the cells at day 4 of differentiation, along with Bodipy staining of intracellular lipid droplets. C/EBPα is nuclear localized in both Dab2 HET and CKO, in contrast to cytoplasmic localization seen for PPARγ in the Dab2 null cells.

Supplemental Figure 5. Impact of Dab2 expression in adipocyte differentiation from mouse embryonic stem cells. Dab2 null (-/-), heterozygous (+/-), and wildtype (+/+) ES cells

were isolated from blastocysts from mating of *dab2* (+/-) mice. The cells were subjected to adipose differentiation following the general protocol: initial differentiation in embryoid bodies and subsequent adherent culturing in lipogeneic induction medium. Dab2-positive wildtype and heterozygous ES cells were efficiently differentiated into adipocytes following the protocol. However, Dab2 null ES cells were severely deficient in adipose differentiation, and few lipid droplet-containing cells were visible following the differentiation procedure after a total of 16 days. The adipocyte differentiation capacity of Dab2-null ES cells is estimated to be 1-2% comparing to Dab2 wildtype or heterozygous ES cells. Representative images of the differentiated cells stained with Oil Red-O to visualize the lipid droplets are shown in two magnifications.

Supplemental Figure 6. Role of Dab2 in adipose differentiation of 3T3-L1 cells. 3T3-L1 pre-adipocyte cells were transfected with shRNA specific to Dab2 or scrambled control, and were subjected to adipogenic differentiation. (**a**) After culturing for 7 days in differentiation medium, the cells were assessed for the degree of adipocyte differentiation by Oil Red-O staining. Representative images are shown. (**b**) The amount of Oil Red-O as an indicator of adipose differentiation was quantified. The differences are statistically significant ($p < 0.005$). (**c**) Cell lysates from undifferentiated (0 day in differentiation medium) and differentiated (7 day in differentiation medium) were analyzed by Western blotting. It is concluded that suppression of Dab2 leads to an increased p-Erk1/2 and subsequent reduced adipose differentiation of 3T3-L1 cells.
